# Health Care as a Team Sport?—Studying Athletics to Improve Interprofessional Collaboration

**DOI:** 10.3390/sports5030062

**Published:** 2017-08-18

**Authors:** Anthony P. Breitbach, Scott Reeves, Simon N. Fletcher

**Affiliations:** 1 Athletic Training Program, Doisy College of Health Sciences, Saint Louis University, St. Louis, MO 63103, USA; 2 Centre for Health and Social Care Research, Faculty of Health, Social Care and Education, Kingston University and St. Georges, University of London, London WC1E 7HU, UK; S.Reeves@sgul.kingston.ac.uk (S.R.); Simon.Fletcher@sgul.kingston.ac.uk (S.N.F.)

**Keywords:** athletics, teamwork, interprofessional practice, world café

## Abstract

Organizations value teamwork and collaboration as they strive to build culture and attain their goals and objectives. Sports provide a useful and easily accessible means to study teamwork. Interprofessional collaborative practice (IPCP) has been identified as a means of improving patient and population health outcomes. Principles of teamwork in sports can inform health professionals and organizations regarding possible improvement strategies and barriers in the optimization of IPCP. Twenty-eight delegates from the 2017 All Together Better Health Conference in Oxford, UK participated in a World Café to discuss the how teamwork in sports can inform IPCP in healthcare and sports medicine. These discussions were captured, transcribed and coded using the domains developed by the Interprofessional Education Collaborative (IPEC) along with extrapersonal or interpersonal loci. Extrapersonal factors regarding structure of leadership, roles and organizational commitment can be positive factors to promote teamwork. However, interpersonal factors affecting communication, values and lack of commitment to collaboration can serve as barriers. Athletic trainers and other sports medicine professionals can serve as valuable members of interprofessional teams and teamwork is essential in the field of sports medicine.

## 1. Background

In 2015, *J. Philos. Sport* published an article entitled “The Nature and Meaning of Teamwork” written by Dr. Paul Gaffney [1]. This article provides a comprehensive review of the teamwork dynamics in sports. Along with Gaffney’s article, the Special Issue of the Journal also included responses by other scholars in the form of commentary articles to Gaffney’s article [2,3,4,5,6,7,8]. Gaffney then wrote a response to the commentary articles [9].

Gaffney recognized the “interpersonal” nature of teamwork as “complex, encompassing many varieties and many dimensions” defining it as “the commitment of individual players to one another and to a common purpose in the context of a shared athletic enterprise” [1]. He states the when the individual players join their teammates and “accept the terms of something like a social contract; joining their purposes together in pursuit of a common goal, pledging to be ‘equal’ in a moral sense, although with non-identical roles” [1].

Cruess and Cruess, when discussing professionalism among health care practitioners, also speak of a “social contract” [10]. This refers to the mutual obligation and expectations that health professionals have with society, which includes their patients, governmental entities and the population overall. A health provider fulfills the societal roles of the “professional” and the “healer”, which are addressed separately in the professional preparation of health care practitioners, but are evaluated together by society [10]. A number of authors writing in healthcare have discussed the role that interprofessional education has on teaching professionalism among health providers [11,12,13,14]. These authors have argued that the traditional notion of professionalism as a singular trait fails to account for the multiple roles a person performs. Thus, it has been recommended that professionalism is better developed in an interprofessional setting as compared to a uniprofessional setting [11].

Gaffney also discusses the “extrapersonal” (macro) elements of teamwork and the influence of team structure on the team dynamics and success of the team. These team members consider this “leap of faith an investment strategy according to which individuals forego immediate satisfactions” to gain the greater satisfaction of success in a team effort [1]. Boxill refers to this as a “social union”,

“We gain respect through our interdependence with each other in a social union, where we recognize we must reciprocate and complement one another, by recognizing differences and how they are essential to a social union, a well-played game, a well-run office, corporation, etc., all displaying teamwork. Athletic teams have coaches who must recognize the roles they are in to make the best decisions. So even if the goal is to give the team the very best chance to win, it is unclear which person you select to join your team, the person you believe is the best athlete or the player who you believe will complement others”.

[2]

Teamwork may seem like a new trend in health care, but interprofessional and collaborative models have been emerging over the last 100 years [15]. Early mentions of these models in 19th century where, in the Boer and Crimean wars, “medicine (and nursing through the influence of (Florence) Nightingale) employed the tactics of their military colleagues, including a chain of command, clear roles and hierarchy of decision making. This approach to organizing care was subsequently transported back to civilian life” [16]. In 1910, Dr. William J. Mayo stated: “The best interest of the patient is the only interest to be considered, and in order that the sick may have the benefit of advancing knowledge, union of forces is necessary” [17]. The World Health Organization provides a definition: “[teamwork] in health-care occurs when multiple health workers from different professional backgrounds provide comprehensive services by working with patients, their families, careers and communities to deliver the highest quality of care across settings” [18].

Effective interprofessional teamwork and collaboration involves the following: (1) optimizes health-services; (2) strengthens health systems; and (3) improves health outcomes [18]. Research evidence has shown that interprofessional practice can improve: access to and coordination of health-services; appropriate use of specialist clinical resources; health outcomes for people with chronic diseases and; patient care and safety. It can also decrease total patient complications; length of hospital stays; tension and conflict among caregivers; staff turnover; hospital admissions; clinical error rates; and mortality rates [18,19,20,21].

Interprofessional Education (IPE) initiatives have been developed to prepare health professionals to be collaborative-ready to work in teams when they enter the workforce [18,22]. In turn, many health professions’ accreditation organizations have updated their standards to include IPE [23]. The National Athletic Trainers’ Association Executive Committee for Education, for example, included IPE as a recommendation its “Future Directions of Athletic Training Education” document in 2012 [24], also advocating for IPE in a white paper: “Interprofessional Education and Practice in Athletic Training” in 2015 [25].

In the effort to provide a foundation for IPE, groups have convened to operationalize collaborative practice using competency-based frameworks. The most prominent of these being the “Core Competencies for Interprofessional Collaborative Practice” introduced by the Interprofessional Education Collaborative (IPEC) in 2010 and revised in 2016 [26]. These competency domains are:

Competency 1: Work with individuals of other professions to maintain a climate of mutual respect and shared values (Values/Ethics for Interprofessional Practice).

Competency 2: Use the knowledge of one’s own role and those of other professions to appropriately assess and address the health care needs of patients and to promote and advance the health of populations (Roles/Responsibilities).

Competency 3: Communicate with patients, families, communities, and professionals in health and other fields in a responsive and responsible manner that supports a team approach to the promotion and maintenance of health and the prevention and treatment of disease (Interprofessional Communication).

Competency 4: Apply relationship-building values and the principles of team dynamics to perform effectively in different team roles to plan, deliver, and evaluate patient/population-centered care and population health programs and policies that are safe, timely, efficient, effective, and equitable (Teams and Teamwork).

There are, however, challenges in translating these educational competencies to health care. First, building interprofessional teams with individuals who work and have been trained in a uniprofessional context makes full adoption of IPCP problematic. Second, translational research in healthcare that shows the connection between IPE and patient outcomes lags behind the progress made in the implementation of IPE and IPCP [15,22]. In addition, there are not yet robust ways to evaluate competency frameworks, which often focus more on individual behaviors and skills or attempt to measure complex tasks with multiple attributes in a single competency/outcome statement [27].

Recent research is beginning to examine collaboration in sports medicine, and is revealing that providing health care in a sporting context can pose additional challenges. In a scoping review of the literature, key issues were identified which have had an effect on collaboration in sports medicine [28]. The following six issues were identified. First, professionalization processes were found to have altered, and in many cases compromised, the influence of sports medicine practitioners, who are often met with more resistance than they would be if they remained casual team members. Second, professional dominance was found to create disparities in power and status between different professional groups (i.e. coaches vs. physicians). Third, status imbalances between sports medicine practitioners created difficulties and friction which restricted their ability to collaborate. Fourth, interprofessional negotiation was found to provide a key mechanism for sports medicine practitioners to try to navigate around tensions related to power/status imbalances. Fifth, ethical behaviors linked to the confidentiality of the patient information in traditional healthcare was found to be more absent in an athletic context as a range of interested parties were expected to be kept fully informed about the athletes’ wellbeing. Finally, issues linked to compromise/competition revealed that sports medicine practitioners needed to balance the desire for performance over care which may mean an athlete misses a sports event; alongside this, there was a distinct competition between sports medicine practitioners which often excluded collaborative input.

In this paper, we present the results of a study that brought together a group of experts in collaborative health care to discuss how teamwork principles from sport could be applied to healthcare and sports medicine.

## 2. Methodology

This study involved the organization of a consensus event using a World Café technique with stakeholders (e.g., practitioners, educators, and researchers) from interprofessional healthcare fields. This project was approved by the Saint Louis University Institutional Review Board (IRB#27224) with letters of collaboration from Kingston University (London, UK) and St. Georges University of London (London, UK) and the All Together Better Health Conference Organizing Committee (Oxford, UK).

### 2.1. Subjects/Participants

This study was undertaken at the “All Together Better Health VIII” (ATBH VIII)—an international conference, which was held at Oxford, United Kingdom in September 2016. It presented an opportunity to engage with clinicians, researchers and educators from many different health professions from many different countries. The ATBH series of conference events are “a platform where practitioners, service users, teachers, managers, policy makers and researchers compare perspectives, exchange experiences and pool resources in response to needs everywhere to effect change, enhance quality and improve safety in care for individuals, families and communities” [29]. 

Twenty-eight participants were recruited voluntarily from the delegates at the ATBH VIII Conference who, by their participation in the conference, demonstrated a strong interest in interprofessional health care. As an international, interprofessional conference, it provided an excellent opportunity to engage a variety of health professions from diverse international contexts. Table 1 lists the professional role and the country reported by the participants in the study. 

### 2.2. Research Design

Drawing on seven integrated design principles, the World Café methodology is a simple, effective, and flexible format for hosting large group dialogue. It applies action research with a participatory approach. It is best used for community development and empowers participants in the discussion of structural inequalities and implications of proposed policy and practices. Investigators should focus more on the development of rich and organic conversation among stakeholders and less on the approval/endorsement of specific policies or legislation [30].

Five components comprise the basic World Café model [31]:

1. Setting: Create a “special” environment, most often modeled after a café. There should be four chairs at each table (optimally)—and no more than five.

2. Welcome and Introduction: The researchers introduce the World Café process, setting the context, sharing the Cafe Etiquette, and putting participants at ease.

3. Small Group Rounds: The process begins with the first of three 10 min rounds of conversation for the small group seated around a table. At the end of the 10 min, each member of the group moves to a different new table.

4. Questions: each round is prefaced with a question specially crafted for the specific context and desired purpose of the World Café.

5. Harvest: After the small groups (and/or in between rounds, as needed), individuals are invited to share insights or other results from their conversations with the rest of the large group.

#### 2.2.1. Data Collection

Participants completed a consent form and table conversations were recorded using handheld digital voice recorders. Each participant received a card that assigned a subject number with table assignments for each round. Participants provided their country and professional role on the reverse side of the card. One participant, selected at random, operated the recorder at each table after being instructed in its use. Each table was also provided with paper “tablecloths” and colored pencils to allow for graphic depictions of the conversations. However, use of the tablecloths was challenging because the conference was not able to provide hard surfaces for this purpose.

The researchers set the stage for the project with a short presentation providing background for the study and explaining the methodology of the study. Then began a series of three, 10 min “rounds” where participants engaged in table conversations on a guiding question. The guiding questions for each round are:

Round 1: “What key features of collaboration, which are emphasized in sport, can be applied to health care?”

Round 2: “What barriers exist to the inclusion of these features in health care?”

Round 3: “How can Interprofessional Collaborative Practice improve sports and exercise medicine at and away from the field/pitch?”

Following the third round, the researchers collected the recorders and the subject cards while reassembling the participants to carry on a “harvest” discussion. There was an attempt to record this harvest conversation, but the voice recorders were not able to capture the discussion adequately. 

At the end of the project, the digital conversations were downloaded onto an encrypted shared drive. That shared drive was accessed by a transcriptionist, who de-identified and transcribed the recordings into documents organized by round number and table number. Several of the recordings where not considered usable due to technical difficulties during the recording process.

#### 2.2.2. Data Analysis

Content analysis was performed on the transcribed and de-identified transcripts using the Dedoose software (SocioCultural Research Consultants LLC, Manhattan Beach, CA, USA) program (http://www.dedoose.com/). Using the software, 45 discrete excerpts were identified with Question 1, and 48 discrete excerpts were identified with Question 2. Each excerpt was thematically coded with one of four IPEC competency domains and either an extrapersonal or interpersonal locus. One of the authors coded each of the excerpts in the first round and these codes were reviewed by the other co-authors in a second round. These authors recommended changes and a final consensus was reached regarding the coded excerpts. Question 3 was not included in the thematic coding because it focused more on specific application strategies in sports medicine.

## 3. Results

Table 2 lists the frequency of codes with the first two questions. In Question 1, which focuses on the positive aspects of teamwork in sports and healthcare, more excerpts were coded with the IPEC domains of Teams/Teamwork (*n* = 18) and Roles/Responsibilities (*n* = 16) than in Question 2. The IPEC domain of Values/Ethics (*n* = 20) was coded more frequently in Question 2, which focuses on the barriers to teamwork in sports and healthcare. Few excerpts were coded with Interprofessional Communication on both questions. The majority of the Question 1 excerpts were coded with an Extrapersonal (*n* = 32) locus and the majority of the Question 2 excerpts were coded with an Interpersonal (*n* = 25) locus.

Table 3 details the interaction between the IPEC and locus coding. Code co-occurrence analysis was performed to assess interaction between the IPEC domain and locus coding. Teams/Teamwork (*n* = 23, 79.31%) and Roles/Responsibilities (*n* = 17, 56.67%) were primarily coded with an Extrapersonal locus. Despite a small number (*n* = 5), Interprofessional Communication had primarily an Interpersonal locus (*n* = 4, 80.00%). The remaining IPEC domain code, Values/Ethics, was nearly equal between the loci.

Table 4 provides representative quotes on Question 1 for each of the IPEC domains along with the Extrapersonal and Intrapersonal loci. The excerpts highlight the structural components of athletics that are not explicitly addressed many times in healthcare. Many of these are benefits involve defined roles on athletic teams. These roles include a clearly defined leader, who is often not expected to be one of the participants in the activity. Another stated benefit involves the physical space and time allocated for practice and team building. Communication and a mutual understanding of goals, especially when defining success, were identified by the participants in their conversations.

Table 5 provides representative quotes on Question 2 for each of the IPEC domains along with the Extrapersonal and Intrapersonal loci. The excerpts in Table 5 highlight the challenges that occur with teamwork in both athletics and healthcare. The majority of these barriers involve and interpersonal locus and the IPEC domains of Communication and Values/Ethics for interprofessional practice. Some of the comments made by the participants also referred to commitment and trust between members of team. 

The table conversations on Question 3 varied in content and theme. Some of the participants stated they were unsure how to answer based on their understanding of sports medicine and how healthcare is provided in that context. Many of the participants mentioned they had limited knowledge of athletic trainers and related professionals such as athletic therapists, sports therapists and kinesiologists. 

Several of the participants had a better sense of the role of athletic trainers in IPCP. One participant talked about the importance of IPE and recognizing common skill sets in the professional preparation curricula:

“My son is a freshman in athletic training, and I run the simulation center at (a university in the United States), and we have athletic training. So I do simulation with those students. But I’m a nurse and I go in and I teach some of their (nursing) courses, like around wound management, because that’s a common skill. But I also work with physical therapists and I work with occupational therapists, so teaching all those common skills to all of those groups in a way that when I talk about wound care they all go oh, it means the same thing, and we’re all going to look at the same thing. Okay, it’s not special just because I have different credentials after my name. So maybe it’s more about do our skill sets, they overlap, they do. I usually identify as common knowledge or shared knowledge, things that we all know together, but maybe it’s less important about the credential, but more important about do our skill sets cover the patient care needs…”

Another participant talked about the importance of recognizing roles and different viewpoints in collaborative healthcare:

“I think part of the collaboration can improve it by having the sport medicine people and athletic therapists, kinesiologists in our case, working together with the nurse practitioners, or working with OT/PT. I know most of our clinics in Canada, will be a PT clinic, but they’ll also have athletic therapy and PT together in a clinic, and so the athletic therapy often does a more immediate, they’re used to the crisis or the intervention on the field where PT are more long term, like stroke rehab and that kind of thing. So I think what you mentioned is really important in that there’s a real benefit, I think there’s an important piece that athletic therapy and kinesiology and just movement professionals bring to the healthcare table that’s actually been missing sometimes in the past.“

Another participant related the importance of including a wide scope of stakeholders on the interprofessional team:

“I do administer an exercise science and athletic training program in the United States, and we, in trying to promote that interprofessional collaborative practice competencies have had to approach this a little differently than our nursing colleagues or our medicine colleagues, because the people we’re collaborating with are not necessarily members of the acute healthcare team, so as you said, it’s helping our students and our clinicians as they’re doing this role, collaborate with teachers and parents and psychologists, you know, sports or whatever, principals or school administration, coaches, I’m just trying to think of the team that kind of surrounds an athlete, at least at the high school and collegiate level, which is where most of our students do their field or practice work. So we’ve spent a lot of time kind of re-conceptualizing who are the different members of the team and what are their roles and responsibilities and how do we communicate and work with them, what are their usual patterns of being...”

## 4. Discussion

This study provided valuable insight, provided by participants with a high level of interest and experience in IPE and IPCP, into issues that health care providers and organizations can learn from the study of athletic organizations. The use of World Café technique generated a unique method of tapping into the shared expertise that exists at a large international conference. Additionally, using the IPEC competency domains and Gaffney’s comparisons of extrapersonal (macro) and interpersonal (micro) aspects of teamwork in athletics in the coding of the excerpts help illuminate the links in collaborative practice(s) between sports and healthcare. 

As presented above, a key finding from this study was that teamwork and collaboration principles from organized athletics can have an influence on interprofessional healthcare practice, and, similarly, healthcare principles can be applied to athletics. Study participants recognized that positive aspects of teamwork in sport that can translate to improvement of care include clarity of purpose/goal, well-defined roles, communication and opportunities for practice and team development. These positive aspects are largely extrapersonal (macro elements) and this may be due to the structure of sport. Many sport organizations have rules and established hierarchies based around teamwork. In many health systems, IPCP developed organically among champions in certain professions and organizations who advocated for a wider adoption of these practices. Additional macro structures such as laws, funding structures and professional jurisdictions can also influence the nature of practice in healthcare.

Participants in the study found that the barriers to collaboration had a higher interpersonal locus. This is understandable because structures are easier to modify than the “hearts and minds” (micro elements) of the persons that contribute to organizational and system culture. They identified interprofessional communication, understanding of role and level of value/commitment/purpose of the stakeholders; in either the athletic or healthcare context. The descriptor “social contract” was also mentioned by Gaffney in sport and Cruess and Cruess in healthcare where team members commit to their role pursuing the common good [1,10]. However, an interprofessional team can be weakened through lack of communication and incongruence of values and ethics toward IPCP and teamwork.

A number of the results presented above support the wider literature related to interprofessional collaboration in sports medicine [28]. The overriding team ethos, which competitive and elite sport engenders, broadly integrates each member of the extended athletic family. There is also evidence to suggest that sports medicine providers can benefit from adopting interprofessional behaviors from conventional healthcare contexts [28]. To fully benefit from the adoption of an athletic teamwork model to encourage collaboration in healthcare; in depth insight into the tensions which sporting networks create; and how professionals respond and adapt to these; will allow a more well-rounded application. Difficulties can arise when applying knowledge from one domain (athletes) to another (healthcare) when there is a lack of understanding of the different contextual factors involved. Therefore, issues such as professionalization, professional dominance, status imbalances, interprofessional negotiation, confidentiality, and compromise and competition [28] need to be paid close attention to when engaging in such translational work.

This study has a number of limitations: (1) the pool of subjects was limited by those who volunteered to participate while attending an international conference; (2) the lack of tables at the study location did not allow for graphic representations on the “tablecloths”; (3) there were user-related technical challenges with the audio recorders in some of the groups rendering those conversations unusable; (4) athletic trainers were not represented among the participants; and (5) the participants in the study may have had limited background in sport. However, despite these limitations the ATBH conference provided an outstanding opportunity to access an international group of health professionals with interest and expertise in interprofessional collaborative practice. Moving forward, in future studies of this type, it would be important to work with the conference organizers to obtain space that includes tables and provide monitors at the tables to give technical assistance to the participants.

## 5. Conclusions

Interprofessional teamwork and collaboration has been identified as a means of improving patient and population health outcomes. Principles of teamwork in sports can inform health professionals and organizations regarding possible improvement strategies and barriers in the optimization of IPCP. Extrapersonal factors regarding structure of leadership and roles, along with organizational commitment can be positive factors to promote teamwork. However, interpersonal factors affecting communication, values and commitment to collaboration can serve as barriers. Athletic trainers and other sports medicine professionals can serve as valuable members of interprofessional teams and teamwork is essential in the field of sports medicine. 

## Figures and Tables

**Table 1:** Participant Demographics (*n* = 28).

Country	Professional Role
Canada	Kinesiologist
Cyprus	Nurse
Denmark	Occupational Therapist
Denmark	Physiotherapist
Germany/Bavaria	Nurse
Indonesia	Medical Doctor
Norway	Pharmacist
Norway	Political Scientist/Social Worker
Norway	Health Promotion/M. Philosophy
Sweden	Nurse
The Netherlands	Education
United Kingdom/Ireland	Medicine/Physician
United Kingdom	Nurse
United Kingdom	Nurse
United Kingdom	Occupational Therapist
United Kingdom	Paramedic
United Kingdom	Physiotherapist
United Kingdom	Physiotherapist
United States	Administration
United States	Artist
United States	Coordinator/Artist
United States	Dentist
United States	Nurse
United States	Nurse
United States	Nurse
United States	Occupational Therapist
United States	Physician
United States	Social Worker

**Table 2:** Code Frequency/Question.

Theme	Q1 (*n* = 45)		Q2 (*n* = 48)	
*IPEC Domain*				
Interprofessional Communication	2	4.44%	3	6.25%
Roles/Responsibilities	16	35.56%	14	29.17%
Teams and Teamwork	18	40.00%	11	22.92%
Values/Ethics for Interprofessional Practice	9	20.00%	20	41.67%
*Locus*				
Extrapersonal	32	71.11%	23	47.92%
Interpersonal	13	28.89%	25	52.08%

**Table 3:** Code Co-Occurrence.

IPEC Domain	Locus				Total	
	Extrapersonal		Interpersonal			
Interprofessional Communication	1	20.00%	4	80.00%	5	5.38%
Roles/Responsibilities	17	56.67%	13	43.33%	30	32.26%
Teams and Teamwork	23	79.31%	6	20.69%	29	31.18%
Values/Ethics for IP Practice	14	48.28%	15	51.72%	29	31.18%
Total	55	59.14%	38	40.86%	93	100.00%

**Table 4:** Question 1: Themes and Excerpts.

What Key Features of Collaboration, Which Are Emphasized in Sport, Can Be Applied to Health Care?	
Codes Applied	Excerpt
Interprofessional Communication, Extrapersonal	Important point is to have time, to have time for communication about the goal, what is the goal, and you need time for training, and you need time probably to go there and work as a team.
Interprofessional Communication, Interpersonal	I just thought communication build into it, certainly the power of the communication between each other would be really key for the healthcare professional.
Roles/Responsibilities, Extrapersonal	A leader, a leader or a team captain is another important thing that we see I think in both sports and healthcare, and situational leadership could be an important thing. Some teams have the same leader all the time, and other teams will have a leader emerge, depending on the situation.
Roles/Responsibilities, Interpersonal	I think clearly defined roles, and especially in teams where everyone’s got that role, everyone knows what they’re doing, and they know where they’re boundaries are, so how you can know which line to go to, which line you can go above.
Teams and Teamwork, Extrapersonal	I would also add that in sports they have a coach that’s external to the team, so if you have a disruption or a hierarchy of players, that the coach, who’s external, is the one that can level the playing field, and that can sort of monitor the interactions, and in healthcare most of the time the coach or the leader of the healthcare team is somebody who’s also a member of the team.
Teams and Teamwork, Interpersonal	A team is motivated to collaborate because it wants to win, and so a healthcare team is motivated to collaborate because it wants to win, but I think the value based question is what does winning mean (in healthcare)?
Values/Ethics for Interprofessional Practice, Extrapersonal	I think sport is more open to new techniques, technology, and also enhancements, whether they’re legal or not, to improve collaborations to make sure the unit works efficiently together. I think sometimes in healthcare we don’t embrace new ideas as quickly, and that’s something that sport certainly does do, and that’s partly because there’s a competitive edge.
Values/Ethics for Interprofessional Practice, Interpersonal	That’s true, and I also think for the collaboration part, because I work in sport somewhat, is the trust and support for each other, respect, all of those things, integrity are pretty much important to teams in a sport, so teamwork applies pretty much support to healthcare teams in my experience.

**Table 5:** Question 2: Themes and Excerpts.

What Barriers Exist to The Inclusion of These Features in Health Care?	
Codes Applied	Excerpt
Interprofessional Communication, Interpersonal	I find in a sports team everyone plays the same sport they are more like they were the same profession, and in healthcare it’s different professionals that might speak different kind of languages, not understanding as well, and it’s more difficult to collaborate because they come from different places.
Roles/Responsibilities, Extrapersonal	Sports teams have this third party person, the coach, because he sits that player who’s out of control and it’s usually the coach who usually says you either get going or you sit down, and I’m going to put somebody else in. But if you’re got a healthcare team and someone’s acting out and you don’t have that third party person, and that person’s sort of the leader, then you have dysfunctionality.
Roles/Responsibilities, Interpersonal	It’s interesting how we would expect a football or basketball player and a tennis player coming together and forming a team. We do expect a doctor and nurse and pharmacist and physical therapist to come together. I think it’s different. I mean a football team is a team of footballers coming together, whereas interprofessional team is an entirely different entity in some ways, so that’s one of the, could be one of the barriers.
Teams and Teamwork, Extrapersonal	Sports teams have this distinct advantage of coming together and practicing over and over again to be a functional team, to the point that the players gradually understand what everybody’s role is and they have the chance to make mistakes in practice, correct those mistakes. They trust that it’s their passing the ball or hitting the ball or doing whatever the sport is, their teammate is going to be where they’re supposed to be, and that they just have the opportunity to learn to work really synergistically and in harmony with each other, which is really different than healthcare. So a barrier in healthcare is that these desperate people come together on a given day or a given shift or in a given hour and are expected to work together, and they may or may not know each other that well, and may or may not have each other’s back in that same way, and may or may not trust that all of this is going to happen. So I think all of those are really elements of what makes this hard to translate into healthcare provision, along with the complexity and the acuity and the speed of which, as healthcare providers you often have to respond to a situation. That just adds another layer of barrier.
Values/Ethics for Interprofessional Practice, Extrapersonal	You might have your neighborhood soccer team that doesn’t have enough kids and they kind of like get things together versus a professional football team, like so, I think that’s a parallel, like we have hospitals that just don’t have enough resources and then hospitals that probably have too many resources.
Values/Ethics for Interprofessional Practice, Interpersonal	With a team, I mean the goal, the mental model is I would think is you want to win, and you want to win as many games as you can, because the more games you win the more money you make probably, and the more notoriety you get. In healthcare I do think we’re kind of bound together with the patient having a good outcome, but I think sometimes we go and your own need to achieve gets in the way, because we really should have a common goal, which is the very best outcome possible for our patients, but I think there’s probably more clarity with the goal in the mental model for sports teams than there may be for our healthcare teams.
